# Protein identification from the parotoid macrogland secretion of *Duttaphrynus melanostictus*

**DOI:** 10.1590/1678-9199-JVATITD-2019-0029

**Published:** 2019-08-19

**Authors:** Douglas Oscar Ceolin Mariano, Marcela Di Giacomo Messias, Patrick Jack Spencer, Daniel Carvalho Pimenta

**Affiliations:** 1 Laboratory of Biochemistry and Biophysics, Butantan Institute, São Paulo, SP, Brazil.; 2 Biotechnology Center, Nuclear and Energy Research Institute (IPEN), São Paulo, SP, Brazil.

**Keywords:** Amphibian skin secretion, Bufonidae, Duttaphrynus melanostictus, Proteomics, Batch chromatography, Asian common toad

## Abstract

**Background:**

Bufonid parotoid macrogland secretion contains several low molecular mass molecules, such as alkaloids and steroids. Nevertheless, its protein content is poorly understood. Herein, we applied a sample preparation methodology that allows the analysis of viscous matrices in order to examine its proteins.

**Methods:**

*Duttaphrynus melanostictus* parotoid macrogland secretion was submitted to ion-exchange batch sample preparation, yielding two fractions: salt-displaced fraction and acid-displaced fraction. Each sample was then fractionated by anionic-exchange chromatography, followed by *in-solution* proteomic analysis.

**Results:**

Forty-two proteins could be identified, such as acyl-CoA-binding protein, alcohol dehydrogenase, calmodulin, galectin and histone. Moreover, *de novo* analyses yielded 153 peptides, whereas BLAST analyses corroborated some of the proteomic-identified proteins. Furthermore, the *de novo* peptide analyses indicate the presence of proteins related to apoptosis, cellular structure, catalysis and transport processes.

**Conclusions:**

Proper sample preparation allowed the proteomic and *de novo* identification of different proteins in the *D. melanostictus* parotoid macrogland secretion. These results may increase the knowledge about the universe of molecules that compose amphibian skin secretion, as well as to understand their biological/physiological role in the granular gland.

## Background

Anuran skin participates in different physiological process and has an important role in chemical defense [1-4]. In these animals, the tegument contains specialized glands, termed granular glands, capable of storing a higher diversity of biological molecules [1, 4-6]. However, some anurans developed glandular accumulation on different body regions. One example is the Bufonidae family, which possess a parotoid macrogland located in the dorsum of the head [7,8].

Bufonid parotoid macrogland secretion contains a wide quantity of alkaloids and steroids. Several studies purified and biologically and/or chemically characterized these molecules on *Bufo* or *Rhinella* species [9-12]. In relation to peptides, Rash et al. [13] identified these molecules in low abundance in *R. marina*. Recently, Huo et al. [14] identified 939 unique peptides in *B. gargarizans* parotoid macrogland secretion by *de novo* approach.

 Proteins are also present in bufonids. Sciani et al. [15] investigated the protein profile of parotoid macrogland secretion of nine bufonids (*Rhinella* sp. and *Rhaebo* sp*.*). The authors showed the presence of several proteins by electrophoresis. Other two researches identified and characterized baserpin and lysozyme from *B. andrewsi* parotoid macrogland secretion [16, 17]. Besides that, proteomics studies performed with *B. bufo*, *B. gargarizans* and *R. schneideri* parotoid macrogland secretion identified 13, 8 and 104 proteins, respectively [14, 18, 19].

In bufonids, protein identification and/or characterization from parotoid macrogland secretion may be limited, mainly because this biological sample is highly viscous, sticky and often water insoluble [15, 20]. Recently, our group developed a methodology to analyze viscous secretions [21]. Employing the ion-exchange batch sample preparation methodology, we were able to biochemically characterize the parotoid macrogland secretion of the Asian common toad *Duttaphrynus melanostictus*, including the retrieval of peptidasic activity, as assessed by zymography [21].

Since our sample preparation step yielded to a protein-rich solution, we performed an *in-solution* proteomics and *de novo* peptide sequencing after anion-exchange batch sample processing of *D. melanostictus* parotoid macrogland secretion.

## Material and Methods

## Reagents

All employed reagents were purchased from Sigma Co. (St. Louis, MO, USA), unless otherwise stated. Amicon Ultra-4 Centrifugal 3 kDa filter and syringe filter (Millex-GV, hydrophobic PVDF 0.22 μm) were purchased from Millipore, USA. pH test strip (pH-Fix 0-14) was obtained from Macherey-Nagel, Germany. QAE-Sephadex A-25 was obtained from Pharmacia Fine Chemicals AB Uppsala, Sweden.

## Skin secretion collection

*D. melanostictus* lyophilized parotoid macrogland secretion was kindly provided by Venom Supplies Pty ltd., Australia.

## Anionic-exchange batch sample methodology

###  Sample preparation 

The material was analyzed according to the protocol developed by Mariano et al. [21] as follows:

*Step 1 - resin preparation:* we resuspended 0.5 g of QAE Sephadex A-25 resin with 12.5 mL of 25 mM ammonium bicarbonate (pH 8.5) for 18h, at room temperature. Then, the tube was centrifuged at 500 g, for 5 min, and the supernatant was discarded. After that, the resin was washed with 12.5 mL 25 mM of ammonium bicarbonate during 30 minutes; after, the tube was centrifuged (500 g, 5 min) and the supernatant was discarded. We repeated this last process twice.

*Step 2 - sample preparation: D. melanostictus* lyophilized parotoid macrogland secretion (~ 100 mg) was resuspended in 20 mL of 25 mM ammonium bicarbonate (pH 8.5) under constant agitation, followed by sonication. We transferred the solution to a tube containing the anionic resin.

*Step 3 - unbound fraction:* we maintained the tube under constant homogenization during 1 h, at room temperature. After that, we centrifuged the tube (500 g, during 5 min), collected the supernatant and termed it as ‘anionic unbound fraction’ (A-UBF). Then, we added 20 mL of 25 mM ammonium bicarbonate (pH 8.5) at the tube and left 1h under constant homogenization. The sample was centrifuged and the supernatant was collected and pooled as A-UBF.

*Step 4 - salt fraction:* following the removal of A-UBF fraction, we added 25 mM ammonium bicarbonate, containing 2 M NaCl (pH 8.5). Here, we conducted this phase as described in step 3: homogenization (1 h) and centrifugation (500 g, during 5 min); however, we collected the supernatant and termed it as ‘anionic salt-displaced fraction’ (A-SDF). This step was repeated twice.

*Step 5 - acid fraction:* finally, we added 25 mM ammonium bicarbonate (pH ~ 3-4). Again, we repeated this step twice: homogenization (1 h), centrifugation (500 g, during 5 min) and supernatant collection [termed as ‘anionic acid-displaced fraction’ (A-ADF)].

A-SDF and A-ADF were mechanically filtered (.22 μm syringe filters) prior to lyophilization.

###  Desalting 

A-SDF and A-ADF were desalted by a HiPrep 26/10 desalting column (GE Healthcare) coupled to an AKTA avant 25 preparative system (GE Healthcare). We resuspended all samples in 5 mL of 25 mM Tris (pH 8.5) and individually loaded into the system. The column eluted at a constant flow rate of 10 mL/min with 25 mM Tris buffer (pH 8.5) and monitored at 220 nm. We collected each peak corresponding to a protein signal and subsequently all samples were lyophilized.

## Chromatographic analysis

Both desalted fractions were concentrated using an Amicon Ultra-4 centrifugal filter (3 kDa), dried, resuspended in 2 mL of 25 mM Tris (pH 8.5) and individually loaded into a Mono Q 5/50 GL column, in a two-buffer system: (1) 25 mM Tris (pH 8.5) and (2) 25 mM Tris, 2 M NaCl (pH 8.5). The column was eluted at a constant flow rate of 1 mL.min^-1^ under a 0 to 50% gradient of buffer 2, during 20 min. We monitored the eluates at 220 nm and automatically collected one mL fractions during the gradient phase.

##  Proteomic analysis: *in-solution* digestion 

We dried aliquots of the A-SDF (1-10) and A-ADF (1- 6) fractions and resuspended each fraction in 8 M urea (100 mM Tris-HCL, pH 8.5) and Tris(2-carboxyethyl)phosphine hydrochloride (TCEP), 5 mM final concentration, for 1h, at room temperature. After that, we added iodoacetamide (IAA) (dissolved in water) (10 mM final concentration) and incubated all samples for 1h, at room temperature and protected from the light. Next, we added 100 mM Tris-HCl (pH 8.5), for urea dilution (2 M final concentration), and 10 µL trypsin (10 ng.µL^-1^ in 100 mM Tris-HCl, pH 8.5) and incubated all samples during 18h, at 30°C. Finally, we stopped the enzymatic reaction adding 50% ACN/5% TFA and dried all samples. Prior to analysis in the mass spectrometer, we used a ZipTip® C-18 pipette tips (Millipore) to desalt and for peptide concentration.

We analyzed all samples in an electrospray ionization quadrupole time-of-flight (ESI-Q-TOF) (Micromass, UK) equipped with binary ultra-performance liquid chromatography system (UPLC) (Acquity, Waters, MA, USA). Samples (5 μL) were separated on a C18 column, using the following mobile phase: (A) 0.1% formic acid (FA) (1:999, v/v) and (B) 0.1% FA in 90% acetonitrile (ACN) (1:900:99, v/v/v). The gradient condition was: 2% B in 0-5 min; 2-40% B in 5-60 min. The mass spectrometry (MS) was equipped with a locked ESI probe and operated in positive mode (ESI+). The electrospray capillary voltage was 3.1 kV, with a cone voltage of 113 V. The cone and desolvation gas flows were 185 and 600 l h−1, respectively. The desolvation temperature was 150°C. MS scans were acquired at 350-1600 mass charge rate (m/z) and MS/MS scans at 50-2000 m/z. The collision energy of the MS/MS analysis was 10-10.6 eV. The software selected automatically ions with a threshold intensity of ≥ 10 for fragmentation.

## Data processing

We loaded and analyzed micromass RAW files by Peaks Studio V7.0 software (BSI, Canada). We adjusted the following parameters for *de novo* peptide sequencing: error tolerance (MS and MS/MS) was set to 0.2 Da; methionine oxidation and carbamidomethylation as variable and fixed modification, respectively; trypsin as cleavage enzyme; and average local confidence (ALC) ≥ 80 %. We performed a basic local alignment search tool (BLAST) with all *de novo* peptides, limiting the search for Amphibia class (taxid: 8292). For a deeper analysis, we only consider alignments presenting the higher scores.

For proteomic identification, we set the following parameters on Peaks software: error tolerance (MS and MS/MS) set to 0.2 Da; methionine oxidation and carbamidomethylation as variable and fixed modification, respectively; trypsin as cleavage enzyme; three maximum missed cleavages; three maximum variable PTMs per peptide; one non-specific cleavage; false discovery rate was ≤ 1 %; and we analyzed all data against Amphibia protein database (167530 entries) (built by retrieving all Uniprot entries associated with this taxon).

## Results

##  Chromatographic analysis of *D. melanostictus* parotoid macrogland secretion after batch sample preparation 

After batch processing, we analyzed A-SDF and A-ADF by anionic-exchange chromatography. According to the chromatographic profile and peak distribution, we obtained a total of 10 and 6 fractions from A-SDF and A-ADF, respectively. The resulting profiles are very similar to those previously obtained. Please refer to Figure 3 in Mariano et al. [21].

## Mass spectrometry analysis

###  Proteomic Identification 

We identified 24 proteins in A-SDF collected fractions (Table 1), being 18 proteins identified in fractions 3-7, after *in solution* digestion. Proteins such as acyl-CoA-binding protein homolog, alcohol dehydrogenase, calmodulin 1, diazepam binding inhibitor, galectin-1, histone H2B and prostaglandin reductase 1 were found in more than one fraction. Only in A-SDF fraction 1 no protein was identified.

**Table 1.** Proteins identified in fractions 1-10 of A-SDF

Fraction	Entry name	Identified protein	Organism	10lgP Score	Peptide	Molecular mass (Da)	PTM	Top BLAST hit
2	ACBP_PELRI	Acyl-CoA-binding protein homolog	*Pelophylax ridibundus*		TKPTDDELKELYGLYK	9808		
					SPQADFDKAAGD(+43.99)VK(+42.01)K		Carboxylation (DKW); Acetylation	
					GLG(sub S)KEDAMSAYVSK		Mutation	
	A4K520_BUFGR	Diazepam binding inhibitor	*Bufo gargarizans*	30.66	GMSKEDAMSAYVSK	9905		
3	C1C3M2_LITCT	NADP-dependent leukotriene B4 12-hydroxydehydrogenase	*Lithobates catesbeiana*	95.25	ASPEGYDC(+57.02)*YFENVGGK	35412		Prostaglandin reductase 1-like [*Nanorana parkeri*]
					IGFDEAFNYK			
	ACBP_PELRI	Acyl-CoA-binding protein homolog	*Pelophylax ridibundus*	51.94	TKPTDDELKELYGLYK	9808		
	F7A8C0_XENTR	Uncharacterized protein (Fragment)	*Xenopus tropicalis*	50.66	IGFDEAFNYK	32004		Prostaglandin reductase 1 *[Xenopus tropicalis*]
					QLLQWVIEGK			
	LEG1_RHIAE	Galectin-1	*Rhinella arenarum*	35.36	LNLKPGHC(+57.02)*VEIK	14711		
					NLNLKPGHC(+57.02)*VEIK			
	AK1A1_XENLA	Alcohol dehydrogenase [NADP(+)]	*Xenopus laevis*	34.37	MPLIGLGTWK	37100		
4	LEG1_RHIAE	Galectin-1	*Rhinella arenarum*	53.51	LNLKPGHC(+57.02)*VEIK	14711		
					NLNLKPGHC(+57.02)*VEIK			
					GFAVNLGEDASNL(sub F)LLHL(sub F)NAR		Mutation	
	ACBP_PELRI	Acyl-CoA-binding protein homolog	*Pelophylax ridibundus*	46.76	TKPTDDELKELYGLYK	9808		
	AK1A1_XENLA	Alcohol dehydrogenase [NADP(+)]	*Xenopus laevis*	44	MPLIGLGTWK	37100		
	A4K520_BUFGR	Diazepam binding inhibitor	*Bufo gargarizans*	21.03	GMSKEDAMSAYVSK	9905		
5	A4K520_BUFGR	Diazepam binding inhibitor	*Bufo gargarizans*	156.1	GMSKEDAMSAYVSK	9905		
					KGMSKEDAMSAYVSK			
					SPQADFDKAAED(+14.02)VKK		Methyl ester	
					ANELIEKH(sub Y)GL		Mutation	
					QSTVGDINIDC(+57.02)*PGMLDLK			
	ACBP_PELRI	Acyl-CoA-binding protein homolog	*Pelophylax ridibundus*	74.97	TKPTDDELKELYGLYK	9808		
	Q4KLC5_XENLA	MGC116485 protein	*Xenopus laevis*	29.86	WEAWNSKK	8374		Acyl-CoA-binding protein homolog [*Xenopus laevis*]
					AKWEAWNSKK			
6	C1C3M2_LITCT	NADP-dependent leukotriene B4 12-hydroxydehydrogenase	*Lithobates catesbeiana*	54.04	ASPEGYDC(+57.02)*YFENVGGK	35412		
					IGFDEAFNYK			
	LEG1_RHIAE	Galectin-1	*Rhinella arenarum*	56.72	NLNLKPGHC(+57.02)*VEIK	14711		
					GFAVNLGEDASNL(sub F)LLH		Mutation	
					GSIPPDC(+57.02)*KGFAVNLGEDASNL(sub F)LLHL(sub F)NAR		Mutation	
					PGHC(+57.02)*VEIK			
	F7A8C0_XENTR	Uncharacterized protein (Fragment)	*Xenopus tropicalis*	40.44	IGFDEAFNYK	32004		Prostaglandin reductase 1 *[Xenopus tropicalis*]
					QLLQWVIEGK			
7	Q641J7_XENTR	Calmodulin 1	*Xenopus tropicalis*	57.87	VFDKDGNGYISAAELR	16838		
	LEG1_RHIAE	Galectin-1	*Rhinella arenarum*	41.85	NLNLKPGHC(+57.02)*VEIK	14711		
					GSIPPDC(+57.02)*KGFAVNLGEDASNL(sub F)LLHL(sub F)NAR		Mutation	
	ACBP_PELRI	Acyl-CoA-binding protein homolog	*Pelophylax ridibundus*	28.9	TKPTDDELKELYGLYK	9808		
8	C1C4P2_LITCT	Calmodulin	*Lithobates catesbeiana*	54.52	SLGQNPTEAELQDMINEVDADGNGTIDFPEFLTMMAR	16838		
					VFDKDGNGYISAAELR			
	LEG1_RHIAE	Galectin-1	*Rhinella arenarum*	51.47	SGDQFSFPVR	14711		
					IVC(+57.02)*NSKEADAWGSEQRE			
					NLNLKPGHC(+57.02)*VEIK			
9	A0A1L8G795_XENLA	Histone H2B	*Xenopus laevis*	34.09	NSFVNDIFER	13935		
10	ACBP_PELRI	Acyl-CoA-binding protein homolog	*Pelophylax ridibundus*	40.54	TKPTDDELKELYGLYK	9808		

*Cysteine carbamidomethylation.

Furthermore, we identified 18 proteins in A-ADF collected fractions (Table 2), being six of them already identified in A-SDF. In the acid fraction, we can highlight the presence of the protein ATP synthase (subunit alpha and beta) and also hemoglobin (subunit beta).

**Table 2:** Proteins identified in fractions 1-6 of A-ADF

Fraction	Entry name	Identified protein	Organism	10lgP	Peptide	Molecular mass (Da)	PTM	Top BLAST hit
1	LEG1_RHIAE	Galectin-1	*Rhinella arenarum*	29.27	NLNLKPGHC(+57.02)*VEIK	14711		
2	A4K520_BUFGR	Diazepam binding inhibitor	*Bufo gargarizans*	117.39	QSTVGDINIDC(+57.02)*PGMLDLK	9905		
					SPQADFDKAAED(+14.02)VKK		Methyl ester	
					ANELIEKH(sub Y)GL		Mutation	
					AKWEAWNS(sub L)KK		Mutation	
					GMSKEDAM(+15.99)^#^SAYVSK			
					KGMSKEDAMSAYVSK			
	ACBP_PELRI	Acyl-CoA-binding protein homolog	*Pelophylax ridibundus*	44.78	TKPTDDELKELYGLYK	9808		
3	A0A1L8H8W7_XENLA	Uncharacterized protein	*Xenopus laevis*	27.03	LVAM(+15.99)^#^GIPESIR	128290		TBC1 domain family member 8-like isoform X2 [*Xenopus laevis*]
	F6U3Y7_XENTR	Myosin IH	*Xenopus tropicalis*	23.29	INSSLANK	120196		
	A0A1L8ESG2_XENLA	Protein Wnt	*Xenopus laevis*	23.27	SSRFSPGTAGRTC(+57.02)*SR	39675		
4	Q7ZWR6_XENLA	ATP synthase subunit beta	*Xenopus laevis*	112.11	866. TVLIMELINNVAK	56374		
					SLQDIIAILGMDELSEEDKLTVSR			
					LVLEVAQHLGES(sub N)TVR		Mutation	
					SLQDIIAILGM(+15.99)^#^DELSEEDKLTVSR			
					KGSITSVQAIYVPAN(sub D)DLTDPAPATTFAHLDATTVLSR		Mutation	
	A0A1L8G6S1_XENLA	Histone H4	*Xenopus laevis*	78.17	AMGIMNSFVNDIFER	26318		
	LEG1_RHIAE	Galectin-1	*Rhinella arenarum*	41.11	GFAVNLGEDASNL(sub F)LLHL(sub F)NAR	14711	Mutation	
				SGDQFSFPVRK				
	Q3KPP1_XENLA	MGC52881 protein	*Xenopus laevis*	35.47	LFIGGLSFETTEESLR	36486		Heterogeneous nuclear ribonucleoprotein A2 homolog 2 isoform X3 [*Xenopus laevis*]
	Q6DD58_XENLA	Tubulin alpha chain	*Xenopus laevis*	33.45	AVFVDLEPTVIDEVR	49887		
6	Q7ZWR6_XENLA	ATP synthase subunit beta	*Xenopus laevis*	134.99	DQEGQDVLLFIDNIFR	56374		
					TVLIMELINNVAK			
					IGLFGGAGVGK			
					FTQAGSEVSALLGR			
					VVDLLAPYAK			
					SLQDIIAILGM(+15.99)^#^DELSEEDKLTVSR			
	Q9I9P5_LITCT	Inner-ear cytokeratin	*Lithobates catesbeiana*	108.48	SLDLDSIIAEVK	56619		
					FLEQQNKVLETK(-.98)		Amidation	
					TP(sub L)NNKFASFIDKVR		Mutation	
	F7CIH4_XENTR	ATP synthase subunit alpha	*Xenopus laevis*	91.29	1059. VLSIGDGIAR	57548		
				EVAAFAQFGSDLDAATQQLLS(sub N)R	Mutation			
				TGAIVDVPVGD(+14.02)ELLGR	Methyl ester			
				TSIAIDTIINQK				
				AVDSLVPIGR				
	G1FF50_9SALA	Beta-actin (Fragment)	*Eurycea cirrigera*	75.81	SYELPDGQVITIGNER	23707		
				TTGIVMDSGDGVTHTVPIYEGYALPHAILR				
				GYSFTTTAER				
	G5DYL7_9PIPI	Tubulin alpha chain (Fragment)	*Hymenochirus curtipes*	58.22	AVFVDLEPTVIDEVR	10921		
	HBB_PELES	Hemoglobin subunit beta	*Pelophylax esculentus*	50.95	LLVVYPWTQR	15424		
	LEG1_RHIAE	Galectin-1	*Rhinella arenarum*	25.59	GFAVNLGEDASNL(sub F)LLHL(sub F)NAR	14711	Mutation	

^#^ Methionine oxidation; *Cysteine carbamidomethylation.

Based on the Gene Ontology (GO) project [22], employing the ‘molecular function’ identifier, we observed that proteins present in A-SDF were associated to binding (four proteins) and/or catalytic activities (seven proteins). While in A-ADF, besides binding activity (12 proteins), we also found proteins classified into nucleoside-triphosphatase activity (five proteins), oxygen carrier activity (one protein), structural molecule activity (three proteins) and transmembrane transporter activity (two proteins).

###  de novo peptides 

This analysis led us to the identification of 102 and 41 different *de novo* peptides in A-SDF and A-ADF collected fractions, respectively (Additional files 1 and 2). Among them, only ten *de novo* peptides were present in both fractions.

BLAST alignment showed that several *de novo* peptides aligned with proteins already identified in our proteomic study, like acyl-CoA-binding protein, alcohol dehydrogenase, galectin and prostaglandin reductase 1 (Additional files 1 and 2, Blast E-value < 0.01, color code: light red). The remaining *de novo* peptides suggest proteins related to: apoptosis (apoptosis regulator Bcl-2-like), binding activity (spectrin beta chain, non-erythrocytic 4; FYN-binding protein-like; FRAS1-related extracellular matrix protein 3), cytoskeleton (keratin; neurofilament light polypeptide), fertilization (zona pellucida sperm-binding protein 4-like), enzymatic activity (hexokinase-2-like; protein ABHD14B-like; serine/threonine-protein kinase akt-1-like; UDP-GlcNAc:betaGal beta-1,3-N-acetylglucosaminyltransferase 9) and transport (golgi to ER traffic protein; large neutral amino acids transporter; apolipoprotein B-100) (Blast E-value < 1, color code: light blue).

It is also important to mention that *de novo* BLAST alignment suggest the presence of the following proteins (Additional files 1 and 2) (Blast E-value > 1, color code: light green): antimicrobial peptide precursor, catechol O-methyltransferase-like, cytochrome P450, estradiol 17-beta-dehydrogenase 12-B-like S, integumentary mucin B.1, metalloproteinase ADAM10-like protein, NADH dehydrogenase subunit 6, N-acetylneuraminate lyase, pappalysin-1, phospholipid-transporting ATPase IC-like, phospholipase A2 crotoxin basic subunit CBb-like, proteasome 26S subunit, protein kinase C delta type-like, protein-tyrosine kinase 2-beta, proteoglycan 4, squalene synthase-like, trans-1,2-dihydrobenzene-1,2-diol dehydrogenase-like and trefoil factor 2-like. We also found uncharacterized proteins in our analysis (Additional files 1 and 2).

## Discussion

Bufonidae is a worldwide amphibian taxon popularly known as the true toads. These anurans store a huge arsenal of bioactive molecules in their parotoid macroglands, such as alkaloids and steroids [5, 10, 12, 23].

However, only a few studies focus on the biochemical and/or biological characterization/identification of proteins in bufonids. One explanation is that the parotoid macrogland secretion exhibit a viscous aspect, which difficult its solubilization. Another point to highlight is the higher quantity of low molecular mass molecules, which makes necessary to prepare properly the sample prior to the protein study.

Huo et al. [14] submitted *B. gargarizans* parotoid macrogland secretion to a cut-off filter (10 kDa). Based on *de novo* sequencing, the authors obtained a < 10 kDa fraction rich in peptides. This fraction exhibited anti-proliferative activity on SMMC-7721 cells under different concentrations. However, the authors do not comment about the presence of low weight molecular mass molecules in < 10 kDa fraction; neither if these molecules were removed nor how they did it.

In another study, Rash et al. [13] submitted *R. marina* parotoid macrogland secretion to two sample preparation steps: dialysis (to remove molecules below 1 kDa) and subsequently, cut-off membrane filter (to remove molecules over 10 kDa and insoluble material). When the authors analyzed the < 10 kDa fraction (termed peptide-enriched sample), they found that the peptide abundance in this material was very low (the authors sequenced only 14 *de novo* peptides). Furthermore, even after dialysis and membrane filter, this material contained high quantities of low molecular mass molecules (< 900 Da).

Utilizing a different approach, Mariano et al. [21] observed a similar result as obtained by Rash et al. [13]. After analyzing *D. melanostictus* parotoid macrogland secretion by batch sample preparation, the authors obtained soluble protein fractions. However, in these soluble fractions they also observed the presence of low molecular mass molecules, however, in low abundance.

The presence of non-protein compounds (alkaloids, biogenic amines, mucus and steroids) interfere in many protein quantification assays [24]. Employing different chromatography strategies (gel filtration, ion exchange and high-performance liquid chromatography), βγ-CAT (a complex of non-lens βγ-crystallin and trefoil factor) [25], lysozyme [17] and KPHTI (a trypsin inhibitor) [26] were purified from *Bombina maxima, B. andrewsi* and *Kaloula pulchra hainana* skin secretion, respectively. Using a similar chromatography steps, Anjolette et al. [24] obtained active protein fractions active on the complement system [24].

Demesa-Balderrama et al. [27] and Cavalcante et al. [28] conducted proteomics studies with *Lithobates spectabilis* and *Dermatonotus muelleri* skin secretion after electrophoresis and classical *in-gel* digestion. However, such approaches are purely analytical and rely on protein separation by electrophoresis, not allowing (typically) protein recovery and/or subsequent biological/biochemical assays. Moreover, the scarce available *omics* databases impair proper proteomic identification.

We employed the ion-exchange batch processing protocol [21] to obtain soluble protein fractions from *D. melanostictus* parotoid macrogland secretion. Following this methodology, we identified by proteomics proteins already described in other studies, such as alcohol dehydrogenase [NADP(+)], calmodulin, galectin, histone H2B and prostaglandin reductase 1 [18, 27-29].

Alcohol dehydrogenase [NADP(+)] is an enzyme belonging to the protein superfamily aldo-keto-reductases (AKRs), responsible for the reduction of aldehydes and ketones to primary and secondary alcohols [30]. Calmodulin is a Ca^+2^-receptor protein involved in signaling pathways, such as growth, metabolic homeostasis, osmotic control, proliferation or reproductive process, through interaction with multiple target proteins [31]. Galectins are a phylogenetically conserved family of lectins involved in different cell signaling pathways, in the immune system, and also in the adult and embryonic tissue development and differentiation [32]. Histones are proteins responsible for the nucleosome structure in eukaryotic cells [33]. Another identified enzyme, prostaglandin reductase 1, is responsible for the irreversible degradation of prostaglandin E and F, leukotriene B4 and lipoxin A4, all endogenous lipid mediators involved in immune response and inflammation, for example [34]. 

In this work, we also identified proteins related to: energy metabolism (ATP synthase subunit alpha and beta), oxygen transport (hemoglobin subunit beta) or structural activity (beta-actin, inner-ear cytokeratin, myosin and tubulin). Furthermore, proteomic analysis revealed the presence of acyl-CoA-binding protein homolog (ACBP) and diazepam binding inhibitor (DBI). Previously, Deng et al. (deposited sequence with no paper associated) found acyl-CoA-binding protein homolog mRNA in *B. garzarians* venom (GenBank: DQ437101.1; ABD75368.1); recently, Huo et al. [14] sequenced *de novo* peptides related to this protein in the aqueous extract of *B. gargarizans* parotoid macrogland secretion.

Studies showed that ACBP and DBI were the same protein [35, 36]. ACBP is highly conserved among different organisms. The literature reports some biological activities of this protein: capacity to displace diazepam from the γ-aminobutyric acid (GABA) receptor in rat brain; to affect the cell growth; to bind to long-chain acyl-CoA esters; to stimulate steroidogenesis in isolated adrenal mitochondria; and to inhibit glucose inducing insulin secretion from pancreas [35, 37]. In addition, after inducing ACBP depletion in a mouse model, animals displayed reduced water content in the intercellular lipid membranes, which led to an elevated transepidermal water loss [38]. In this way, ACBP may act/help on physiological process that happens in anuran skin, once this organ is involved in gas-exchange, ionic and osmotic regulation, protection and thermoregulation [1-4]. The presence of steroid molecules in bufonid parotoid macrogland secretion [9-12, 15] may suggest the involvement of ACBP in the synthesis or transport of such molecules.

Moreover, we identified proteins involved in housekeeping function. Sousa-Filho et al. [18] found a similar protein profile in *R. schneideri* parotoid macrogland secretion (proteins related to carbohydrate metabolism, cell matrix, lipid metabolism, protein metabolism or uncharacterized proteins). Kowalski et al. [19] identified proteins involved in the antioxidant system (phospholipid hydroperoxide glutathione peroxidase), apoptosis (serine-threonine kinase), energy metabolism (muscle creatine kinase) or protein recycling (proteasome subunit α type-7-A) from *B. bufo* parotoid macrogland extract. The authors also identified a serine peptidase (snake venom serine protease homolog) that may exert toxic activity [19]. Rash et al. [13] suggested that the *de novo* peptides obtained from *R. marina* parotoid macrogland secretion were breakdown products of proteins involved in cell maintenance.

Each gland that composes the parotoid macrogland is a syncytial cell filled by cytoplasm. Several nuclei are present on the periphery of the gland, as well as organelles (Golgi stacks, mitochondria and rough endoplasmic reticulum). In a central position, we found several granules [7, 8]. Upon mechanical pressure, the secretion expels/ejects much like a champagne cork. Therefore, cellular components can be expelled together with the secretion, like the cell machinery, cytoplasm, whole organelles and eventually nuclei [39]. Therefore, it was not unusual to identify house-keeping proteins in bufonid parotoid macrogland secretion.

*De novo* peptide sequencing relies on the determination of a peptide sequence directly from the mass spectrometry data, without the aid of a protein database [40]. Using this rationale, we deduced circa 150 *de novo* peptides from SDF and ADF fractions. After performing a BLAST search against Amphibia database, we proposed another dataset of proteins related to: binding, enzymatic activity or molecular transport (Additional files 1 and 2).

Furthermore, there were other interesting protein possibilities derived from that approach. However, due to the poor aligned E-value (but not a low ALC score) we will not discuss deeply these results, avoiding too much speculation. The only protein that we would call the attention is the antimicrobial peptide precursor (Additional file 1: fractions 3 and 4; Additional file 2: fraction 5). Recently, Shibao et al. [41] identified several classes of antimicrobial peptides in *Rhinella schneideri* skin glands by transcriptomic analysis. However, complementary studies are still necessary to confirm the actual existence of them.

Demesa-Balderrama et al. [27] performed an *in-gel* digestion and *de novo* peptide sequencing to study proteins from *Lithobates spectabilis* skin secretion. The authors found 111 *de novo* peptides, identifying 15 proteins (E-value < 0.077). Nonetheless, their research discuss about the aspects that we must considerer when performing protein identification based on *de novo* peptides. One of them is the decreased number of amphibian skin proteins deposited in public data banks. Such problematic was also observed by Souza-Filho et al. [18] and in the present study, supporting the need for transcriptome studies with amphibian granular gland.

The IEX batch sample preparation led to the identification of 42 proteins in *D. melanostictus* parotoid macrogland secretion. These results may help to increase the knowledge on the amphibian skin secretion composition, as well as to infer possible biological activities exerted by these proteins. *De novo* peptide sequencing and subsequent BLAST alignment suggest a complementary protein dataset in *D. melanostictus* parotoid macrogland secretion.

## >Abbreviations

SDF: salt-displaced fraction; ADF: acid-displaced fraction; A-UBF: anionic unbound fraction; A-SDF: anionic salt-displaced fraction; A-ADF: anionic acid-displaced fraction; TCEP: Tris(2-carboxyethyl)phosphine hydrochloride; IAA: iodoacetamide; ESI-Q-TOF: electrospray-quadrupole-time of flight; UPLC: ultra-performance liquid chromatography system; FA: formic acid; CAN: acetonitrile; MS: mass spectrometry; ALC: average local confidence; AKR: aldo-keto-reductase; ACBP: acyl-CoA-binding protein homolog; DBI: diazepam binding inhibitor; GABA: γ-aminobutyric acid

## Supplementary material

The following online material is available for this article:

Additional file 1.
*de novo* peptides identified from *D. melanostictus* A-SDF.

Additional file 2.
*de novo* peptides identified from *D. melanostictus* A-ADF.
